# Supplementary material for: Socioeconomic disadvantage as a driver of non-urgent emergency department presentations: A retrospective data analysis
Source: PLoS One. 2020 Apr 13;15(4):e0231429. doi: 10.1371/journal.pone.0231429 (PMC7153867; doi:10.1371/journal.pone.0231429)
Supplement: S1 File — (DOCX) [file pone.0231429.s001.docx]

**Supporting Information:** Socioeconomic disadvantage as a driver of non-urgent emergency department presentations: A retrospective data analysis

1. ABS. 2016 Census DataPacks. Canberra: Australian Bureau of Statistics; 2017 Canberra: Australian Bureau of Statistics; [Available from: <https://datapacks.censusdata.abs.gov.au/datapacks/>.

2. ABS. 2011 Census DataPacks. Canberra: Australian Bureau of Statistics; 2012 Canberra [Available from: <https://datapacks.censusdata.abs.gov.au/datapacks/>.
